# Paeoniflorin mitigates PBC-induced liver fibrosis by repressing NLRP3 formation

**DOI:** 10.1590/ACB361106

**Published:** 2022-02-18

**Authors:** Yizhe Zhang, Shujie Zhang, Xin Luo, Han Zhao, Xiaoxing Xiang

**Affiliations:** 1 PhD. College of life Science - Zhengzhou University - Zhongyuan District - Henan Province, China.; 2 MS. College of life Science - Zhengzhou University - Zhongyuan District - Henan Province, China.; 3 MS. College of life Science - Zhengzhou University - Zhongyuan District - Henan Province, China.; 4 BA. College of life Science - Zhengzhou University - Zhongyuan District - Henan Province, China.; 5 PhD. Department of Gastroenterology - Taizhou people’s Hospital affiliated to Medical College of Yangzhou University - Jiangsu, China.

**Keywords:** Liver Cirrhosis, Biliary, NLR Family, Pyrin Domain-Containing 3 Protein, Liver Cirrhosis, Mice

## Abstract

**Purpose:**

To delve into the influence of paeoniflorin (PA) on abating primary biliary cholangitis (PBC)-induced liver fibrosis and its causative role.

**Methods:**

Our team allocated the mice to control group, PA group, PBC group and PBC+PA group. We recorded the weight change of mice in each group. We used Masson staining for determining liver fibrosis, immunofluorescence staining for measuring tumor necrosis factor-α (TNF-α) expression, quantitative real-time polymerase chain reaction (qRT-PCR) for assaying related gene expression, as well as Western blot for testing related protein expression.

**Results:**

The weight of PBC model mice declined. Twenty-four weeks after modeling, the positive rate of anti-mitochondrial antibody-M2 (AMA-M2) in PBC mice reached 100%. Alkaline phosphatase (ALP), alanine aminotransferase (ALT), aspartate aminotransferase (AST), hydroxyproline (HYP), laminin (LN), procollagen type III (PC III), and malondialdehyde (MDA) contents saliently waxed (p<0.01). Meanwhile, superoxide dismutase (SOD) and glutathione peroxidase (GSH-px) activity patently waned (p<0.01). Liver fibrosis levels were flagrantly higher (p<0.01), and TNF-α, NOD-like receptor protein 3 (NLRP3), caspase-1, interleukin-18 (IL-18), and interleukin-1β (IL-1β) protein or gene expression were manifestly up-regulated (p<0.01). PA could restore the weight of PBC mice, strikingly restrain the positive expression of AMA-M2, and down-regulate serum ALP, ALT, AST, HYP, LN, PC III, MDA in PBC mice (p<0.01). PA could also significantly up-regulate SOD and GSH-px levels (p<0.01), down-regulate IL-1β, IL-18, caspase-1, NLRP3, and TNF-α protein or gene expression in PBC mice (p<0.01) and inhibit liver fibrosis levels (p<0.01).

**Conclusions:**

PA can reduce PBC-induced liver fibrosis in mice and may function by curbing the formation of NLRP3.

## Introduction

Paeoniflorin (PA), namely total glucosides of peony (TGP) capsule, is mainly utilized for remedying rheumatoid arthritis, rheumatism, and bone injuries. TGP is a powdered substance extracted from the root of peony. As a Chinese herbal medicine, it has been adopted for assuaging pain and it is one of the effectual prescription drugs for treating liver disease. Studies have authenticated that TGP is efficacious against immune liver fibrosis^1^, colitis^2^, Sjogren’s syndrome^3^, immune encephalomyelitis^4^, rheumatoid arthritis^5^, systemic lupus erythematosus^6^, etc.

Primary biliary cirrhosis (PBC) is a chronic cholestatic liver disease of autoimmune origin, with female dominance, high serum anti-mitochondrial antibody (AMA), anti-nuclear antibodies (ANAs) expression and autoimmune-mediated liver cirrhosis as characteristics^7^. Common histopathological feature of fibrotic diseases is the exceeding accumulation of extracellular matrix, which destroys the normal structure of physiological tissues^8^. Tissue fibrosis can happen after various stimuli and it is accompanied by subsequent inflammation^9^. Liver fibrosis is a gradual pathological process in which matrix protein excessively deposits, leading to the final accumulation in the extracellular space^10^^,^ 11. Chronic damage can lead to liver fibrosis, which is a physiological response to multiple injuries, embracing viral hepatitis, excessive drinking, drugs, metabolic illnesses, etc. If this damage is joined with invalid regeneration and restoration, the aberration of normal liver structure will increase, and finally result in liver cirrhosis. Studies have demystified that liver fibrosis and even cirrhosis are dynamic processes, with two stages of progression and regression^12^^,^ 13. Hence, comprehending the process of fibrosis and the early accumulation of scars is conducive to seek for therapeutic targets that can slow the development of liver fibrosis, as well as approaches that can lessen matrix synthesis or augment matrix degradation^14^.

Hereby, we evaluated the influence of PA on PBS-mediated liver fibrosis and its associated mechanism of action, aiming to better apprehend the causative role of PA alleviating liver fibrosis.

## Methods

The experiment was approved by the Ethics Committee of College of Life Science, Zhengzhou University (No. 2033).

### Materials

The materials used in this study were mice, PA [purity: 98.78% (HPLC), Ningbo Liwah Pharmaceutical Co., Ltd.], polyinosinic–polycytidylic acid (polyI: C, Sigma), malondialdehyde (MDA) detection kit, superoxide dismutase (SOD) activity detection kit, reduced glutathione (GSH) detection kit (Solarbio), enzyme-linked immunosorbent assay (ELISA) kit (TSZ), tumor necrosis factor-α (TNF-α), NOD-like receptor protein 3 (NLRP3), interleukin-18 (IL-18), caspase-1, interleukin-1β (IL-1β), and β-Actin antibodies (Abcam).

### Mouse grouping and PBC model preparation

After one week of adaptation, female mice (6-8 weeks old) in PBC group (n=24) were intraperitoneally injected with 5 mg/kg polyI: C (1 mg/mL), twice a week, lasting 24 weeks. Mice in PBC+PA group (n=24) were injected with polyI: C and were given PA (50 mg/kg) by gavage daily. Mice in control group (n=24) received equivalent normal saline via intraperitoneal injection. Mice in PA group (n=24) received PA (50 mg/kg) by gavage daily.

### Record of general status and body weight

Our team observed and recorded the mental state, diet and weight change of each group of mice daily and drew a weight change curve.

### ELISA

Blood of each group was collected from the eyeball at each time period. After the serum was separated, ELISA kit was utilized for examining serum anti-mitochondrial antibody-M2 (AMA-M2), alkaline phosphatase (ALP), alanine aminotransferase (ALT), aspartate aminotransferase (AST), and hydroxyproline (HYP) and evaluating related protein expression for each group. The detailed operation was proceeded following the kit’s recommendation.

### Detection of HYP content

After sacrifice of mice, we accurately weighed the wet weight of liver tissue. After homogenization, we calculated the HYP content in liver tissue of mice in each group, following the Equation 1:


HYP (mg/g) = (absorbance of tested sample – absorbance of blank sample) / (absorbance of standard sample = absorbance of blank sample) × standard HYP (5 mg/L) × total volume of hydrolysate (5 mL) / wet weight of liver tissue (mg) (1)

### Detection of MDA content, SOD, and GSH-px activity

After sacrifice of mice, we accurately weighed the wet weight of liver tissue. Later, we added Tris-HCl (pH=7.4) to homogenize the liver tissue at 4°C. After centrifugation at 1,000 r/min lasting 10 min, the supernatant was immediately adopted for MDA, SOD, and glutathione peroxidase (GSH-px) determination. MDA determination was executed by means of thiobarbituric acid method. Total SOD and GSH-px measurement was based on their potency to suppress the oxidation of hydroxylamine by the xanthine-xanthine oxidase system and analyzed by commercial diagnostic kits following the manufacturer’s instructions. The absorbance of each well was determined by a microplate reader, and the activity was calculated according to a formula.

### Masson staining

After sacrifice of mice, our staff washed the livers three times in cold normal saline, and fixed liver tissues nightlong utilizing 10% formalin fixative solution. After washing, liver tissues were embedded in paraffin. Immunohistochemical analysis was performed using the following steps: paraffin sections were deparaffinized and hydrated. Tissue sections were placed in citric acid buffer (pH 6) for antigen retrieval in a microwave oven. We implemented Masson staining and observed the pathological changes of the liver exploiting microscope.

### Immunofluorescence staining

After deparaffinization and rehydration of mouse liver sections, our staff soaked the specimens in PBS involving 0.3% Triton lasting 30 min, blocked the sections using 10% goat serum, and added TNF-α to incubate overnight at 4°C. After washing, we added the diluted fluorescent secondary antibody dropwise, incubated it in a humid box at indoor temperature lasting 1 h, added DAPI dropwise and incubated it in the dark lasting 5 min and observed it through a fluorescence microscope.

### qRT-PCR

After sacrifice of mice, we hastily took out the liver tissues and preserved them in liquid nitrogen. An appropriate number of liver tissues were also grounded in liquid nitrogen. Afterwards, we added 1 mL TRIzol to mix and let stand lasting 5 min, added 200 μL chloroform and shook it. Centrifugation was completed at 4^o^C and 12,000 rpm lasting 10 min. We took supernatant, added an equal volume of isopropanol, left it at indoor temperature lasting 10 min, centrifuged it at 4 °C and 12,000 rpm lasting 15 min, washed the precipitate twice with freshly prepared 75% ethanol, added an appropriate amount of diethylpyrocarbonate (DEPC) water to dissolve and detected concentration by NanoDrop spectrophotometer. Primers were devised online (www.ncbi.nlm.nih.gov/tools/primer-blast/) and synthesized by Shanghai Sangon Biotech Co., Ltd. cDNA synthesis was achieved adopting RT Master Mix kit. Applying SYBR-Green real-time polymerase chain reaction (PCR) master mix, quantitative RT-PCR (qRT-PCR) was fulfilled according to ABI 7500 sequence detection system. Using cycle threshold (Ct) value, we appraised the transcription level. Employing ^2-ΔΔCt^ method, we acquired the target amount normalized to the endogenous reference (Table 1).

**Table 1:** Primer sequences of NLRP3, caspase-1, IL-18, IL-1β and GAPDH.

**Primer**	**Sequence**
NLRP3 F	5’- CCAAGGCTGCTATCTGGAGG-3’
NLRP3 R	5’- TTGCAACGGACACTCGTCAT-3’
Caspase-1 F	5’- CTGGGACCCTCAAGTTTTGC-3’
Caspase-1 R	5’- AGACGTGTACGAGTGGTTGT-3’
IL-18 F	5’- AACACTGGCTGTTCCCACAA -3’
IL-18 R	5’- CGGGGCCTGAGGATTATAGC -3’
IL-1β F	5’- TGCCACCTTTTGACAGTGATG -3’
IL-1β R	5’- CAAAGGTTTGGAAGCAGCCC -3’
GAPDH F	5’- CCCTTAAGAGGGATGCTGCC -3’
GAPDH R	5’- TACGGCCAAATCCGTTCACA -3’

NLRP3: NOD-like receptor protein 3; IL-18: interleukin-18; IL-1β: interleukin-1β; GAPDH: glyceraldehyde 3-phosphate dehydrogenase

### Western blotting

After sacrifice of mice, our crew rapidly took out the liver tissue, lysed it overnight at 4°C with cell lysate, extracted the total protein by centrifugation at 13,000 rpm and determined protein concentration by bicinchoninic acid (BCA) protein assay. Based on the biuret reaction, protein reduces Cu^2+^ to Cu^+^ in an alkaline environment, producing a purple-blue complex with a high absorbance value at 562 nm. The amount of the reaction product was proportional to the protein concentration. We separated the proteins via 8% SDS-PAGE and transferred them to polyvinylidene fluoride (PVDF) membrane. Subsequently, we blocked the membrane with 5% skimmed milk powder, as well as 0.1% Tween-20 in Tris buffered saline, then we incubated it with primary antibodies NLRP3, caspase-1, IL-18, IL-1β and β-Actin overnight at 4 °C with gentle shaking. After reaction with primary antibody, it was incubated with horseradish peroxidase (HRP)-labeled second antibody overnight. Ultimately, we applied enhanced chemiluminescence (ECL) reagent to detect protein.

### Statistical analysis

We statistically processed the data which were exhibited as mean± standard deviation (SD) by use of Statistical Package for the Social Sciences (SPSS) 20.0 software. Data between groups were compared by one-way analysis of variance (ANOVA) followed by Tukey’s post hoc tests. Also, we employed independent sample t test to implement inter-group comparison. Significance is indicated as follows: p<0.05.

## Results

### Alterations in body weight and physical condition of mice

**Figure 1:**
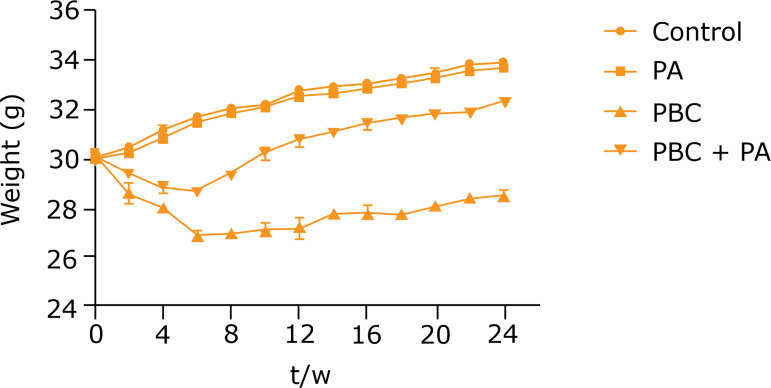
Changes in body weight of mice in each group.

PA: paeoniflorin; PBC: primary biliary cholangitis.

Before modeling, mice in each group had good quality of life and mental status, and normal diet. After modeling, mice in PBC group and PBC+PA group showed changes in life and mental status, reduced activity and diet and lost weight. Among them, PBC group harbored lower body weight than PBC+PA group. Mice in control group and PA group were in the same state as before modeling, and their body weight gradually increased (Fig. 1).

### Impact of PA on serum ALP, ALT, and AST contents in mice

**Figure 2:**
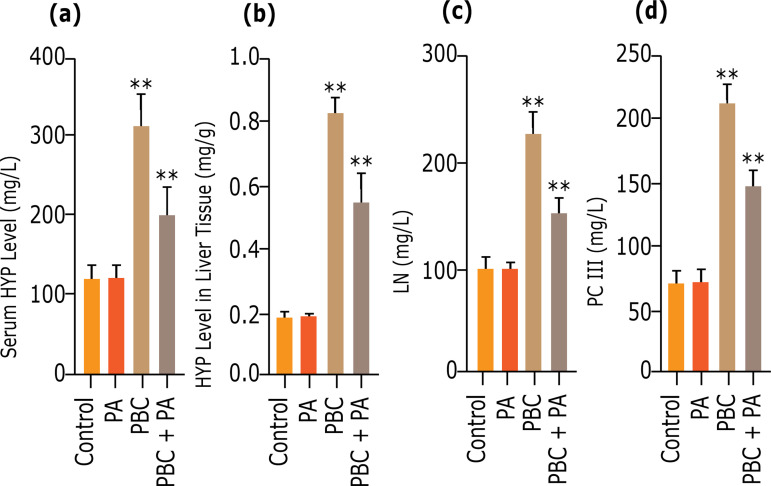
**(a)** Serum ALP, **(b)** ALT and **(c)** AST contents after 24 weeks.

PA: paeoniflorin; PBC: primary biliary cholangitis; ALP: alkaline phosphatase; ALT: alanine aminotransferase; AST: aspartate aminotransferase; **p<0.01.

Serum ALP, ALT and AST contents after 24 weeks in control group and PA group were not brilliantly different, but they were pronouncedly lower than those in PBC group and PBC+PA group (p<0.01). Serum ALP, ALT and AST contents in PBC group were the highest, which were overtly higher than those in PBC+PA group (p<0.01) (Fig. 2).

### Detection of fibrosis indicators in mice

**Figure 3:**
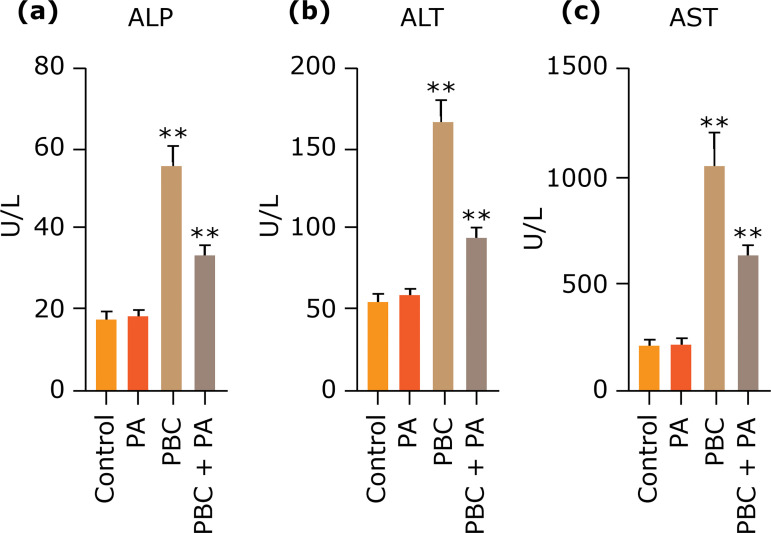
Liver fibrosis indicators after 24 weeks. **(a)** Serum HYP level; **(b)** liver HYP level; **(c)** serum LN level; **(d)** serum PC III level.

PA: paeoniflorin; PBC: primary biliary cholangitis; HYP: hydroxyproline; LN: laminin; PC III: procollagen type III; **p<0.01.

Twenty-four weeks after modeling, serum HYP, LN, PC III, and liver tissue HYP contents in control group and PA group were all lower expression levels, which were transparently lower than those in PBC and PBC+PA group (p<0.01). Serum HYP, LN, PC III, and liver tissue HYP contents in PBC group were the highest, which were eminently higher than those in PBC+PA group (p<0.01) (Fig. 3).

### Measurement of MDA content and SOD and GSH-px activity

**Figure 4:**
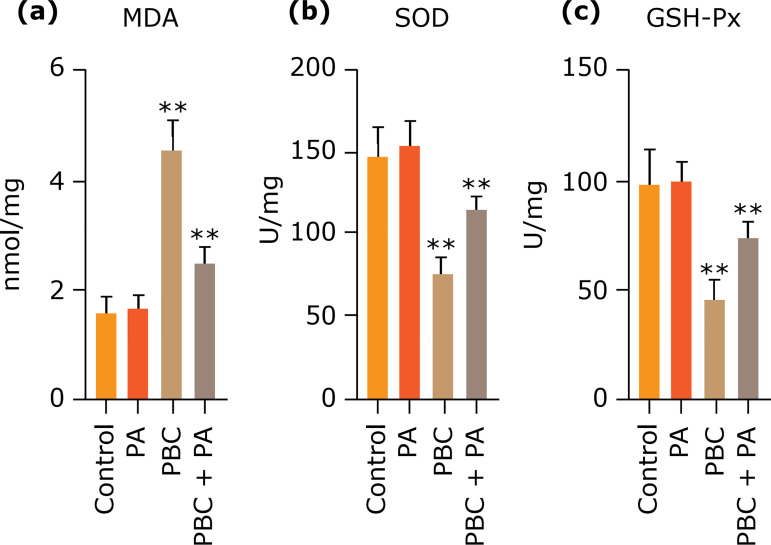
**(a)** MDA content and **b)** SOD, **(c)** GSH-px activity in mouse liver tissue after 24 weeks.

PA: paeoniflorin; PBC: primary biliary cholangitis; MDA: malondialdehyde; SOD: superoxide dismutase; GSH-Px: glutathione peroxidase; **p<0.01.

PBC group held prominently higher MDA content in mouse liver tissue than other groups (p<0.01), whereas PBC+PA group harbored preeminently lower MDA content than PBC group (p<0.01). PBC group owned visibly lower GSH-px/SOD activity in liver tissue than other groups (p<0.01), whereas PBC+PA possessed observably higher GSH-px/SOD activity than PBC group (p<0.01) (Fig. 4).

### Masson staining of mouse liver tissue

**Figure 5:**
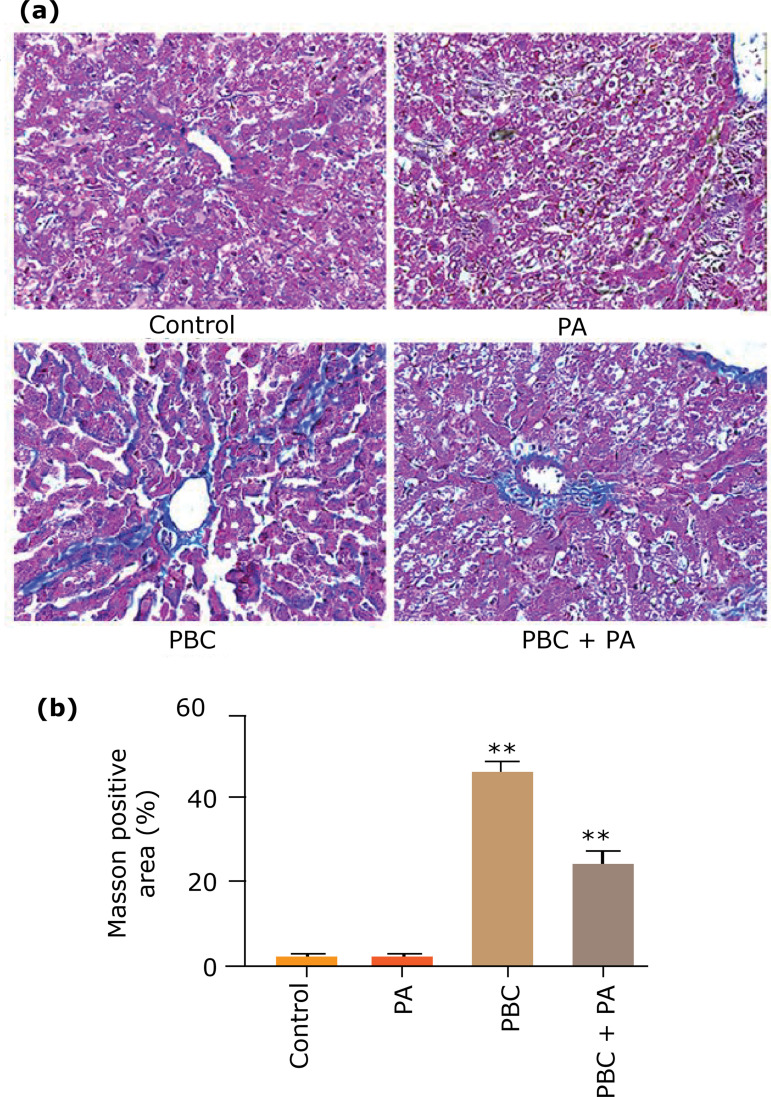
Masson staining detects fibrosis degree in the liver tissue after 24 weeks. **(a)** Masson staining picture (x200); **(b)** positive staining area.

PA: paeoniflorin; PBC: primary biliary cholangitis; **p<0.01.

Masson staining uncovered that after 24 weeks. The liver tissues of the control group and PA group were good with less fibrosis, while the fibrosis around the small bile duct in PBC group was obvious; the liver tissue of the PBC+PA group was partially fibrotic (Fig. 5a). Masson positive area evinced that fibrosis area in PBC group was the largest, which was sensibly higher than that in PBC+PA group (p<0.01). PBC+PA group held noticeably larger fibrosis area than control group and PA group (p<0.01) (Fig. 5b).

### TNF-α expression in mouse liver tissue

**Figure 6:**
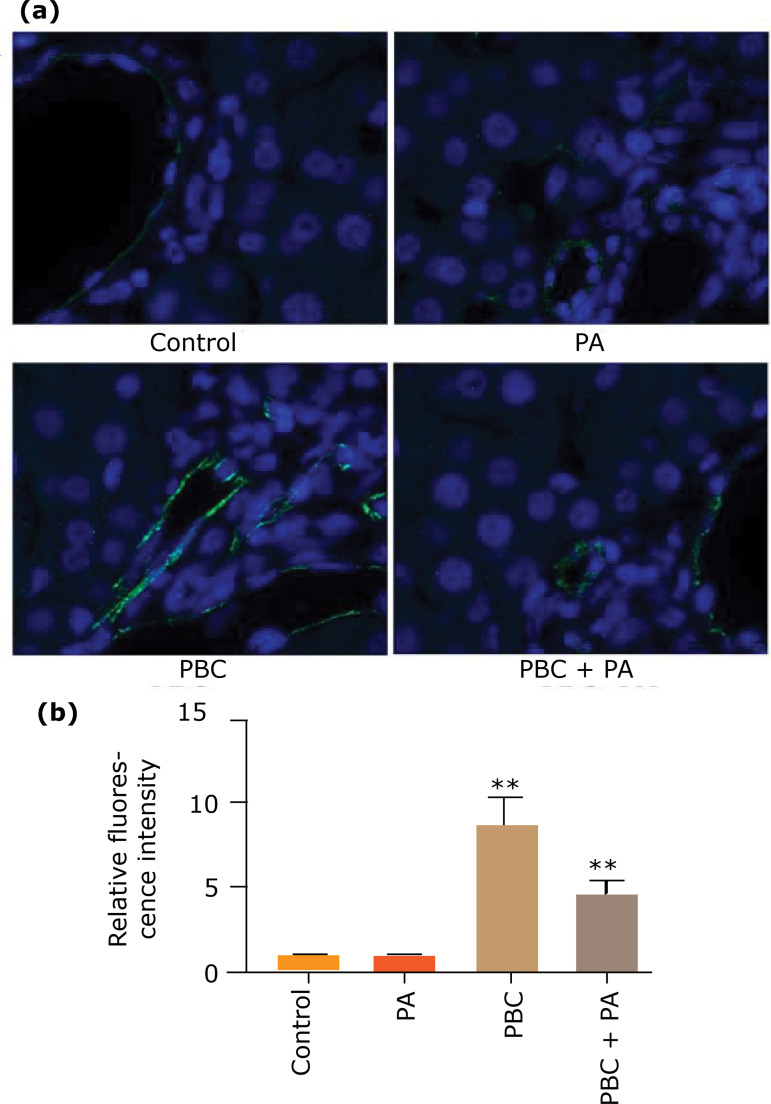
Immunofluorescence staining to prospect TNF-α expression in the liver tissue after 24 weeks. **(a)** Immunofluorescence cell staining; **(b)** relative fluorescence intensity.

PA: paeoniflorin; PBC: primary biliary cholangitis; TNF- α: tumor necrosis factor-α; **p<0.01.

Immunofluorescence staining displayed that after 24 weeks. TNF-α contents in liver tissue of PBC group was strongly expressed in the portal vein infiltration site, and PBC group owned markedly higher fluorescence intensity than other groups (p<0.01). Fluorescence intensity of PBC+PA group was notably weaker than that of PBC group (p<0.01), but stronger than that of control group and PA group (p<0.01) (Fig. 6).

### Influence of PA on inflammasome NLRP3 gene expression

**Figure 7:**
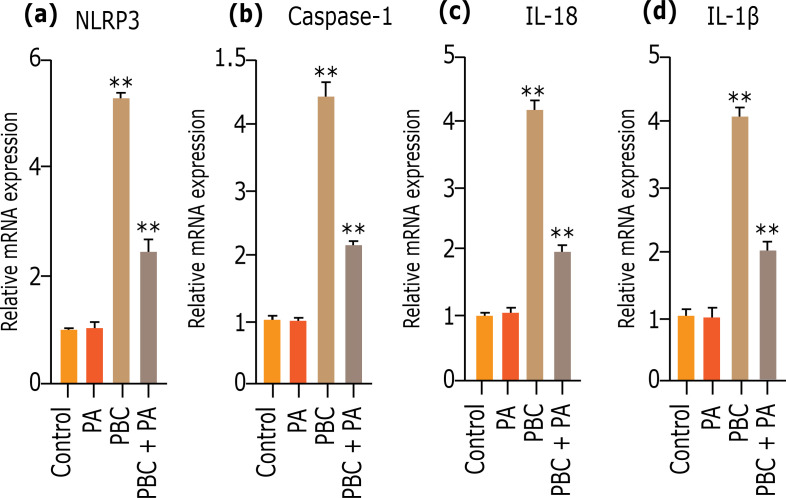
qRT-PCR detects **(a)** NLRP3, **(b)** caspase-1, **(c)** IL-18, and **(d)** IL-1β mRNA expression in mouse liver tissues after 24 weeks.

QRT-PCR detecting mRNA expression in mouse liver tissues after 24 weeks uncloaked that PBC group possessed conspicuously higher and NLRP3, caspase-1, IL-18 and IL-1β mRNA levels than other groups (p<0.01). In PBC+PA group, PA treatment conspicuously reduced NLRP3, caspase-1, IL-18 and IL-1β mRNA levels in PBC model mouse (p<0.01) (Fig. 7).

### Influence of PA on inflammasome NLRP3 protein expression

**Figure 8:**
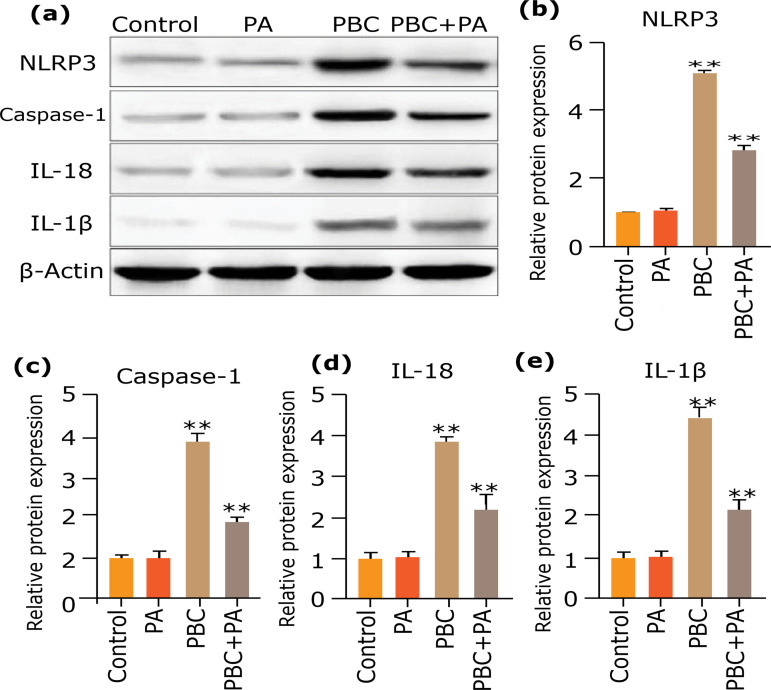
Western blot determines protein levels in mouse liver tissue after 24 weeks. **(a)** Western blot diagram; **(b)** NLRP3 relative expression; **(c)** caspase-1 relative expression; **(d)** IL-18 relative expression; **(e)** IL-1β relative expression.

PA: paeoniflorin; PBC: primary biliary cholangitis; NLRP3: NOD-like receptor protein 3; IL-18: interleukin-18; IL-1β: interleukin-1β; qRT-PCR: quantitative real-time polymerase chain reaction; **p<0.01.

Western blot detecting protein levels in the liver tissue of mice after 24 weeks evinced that PBC group harbored perspicuously higher NLRP3, caspase-1, IL-18 and IL-1β protein expressions than other groups (p<0.01). In PBC+PA group, PA treatment remarkably dwindled IL-1β, IL-18, caspase-1, and NLRP3 protein expressions in PBC model mice (p<0.01) (Fig. 8).

## Discussion

PolyI: C, a type I interferon, can be injected into mice to establish PBC animal models (15). After injection of polyI: C, a slight increase in serum ALT/AST can be observed, and the liver develops mild inflammation and focal necrosis (16). We prepared mouse PBC animal model successfully by using polyI: C. The liver tissue showed inflammatory infiltration, weight loss, and increased serum ALP, ALT and AST, indicating that liver damage occurred. Additionally, LN, as well as PC III, is excellent serum hallmark of liver fibrosis. Elevated liver HYP and serum LN, PC III levels hinted that collagen was precipitated in liver tissue, and liver fibrosis occurred. The enhancement of MDA value and reduction of GSH-px/SOD activity demonstrated that oxidation response occurred, and liver metabolism was impaired. Serological marker of PBC disease is the presence of AMA positive expression^17^. After we injected polyI: C, the AMA positive rate reached 100%, indicating that the PBC mouse model was successfully modeled.

The cardinal active ingredient of PA is TGP. Studies have authenticated that TGP harbors anti-inflammatory, antioxidant, hepatoprotective, and immunomodulatory activities without obvious toxicity or adverse reactions^18^. TGP down-regulates serum ALT/AST levels by repressing the increase of ALT/AST in CCl4-mediated liver damage, indicating that TGP meliorates the structural completeness of liver cell membranes, thereby safeguarding mice from CCl4-induced hepatotoxicity^19^. Wang *et al*.^15^ have substantiated that TGP ameliorates human albumin-induced liver structural change, diminishes lobular necrosis, and dramatically minifies collagen content, and it has a beneficial effect on liver fibrosis in rats by restraining collagen synthesis, as well as reducing oxidative stress. Our findings exhibited that PA can attenuate the high-positive rate of AMA-M2 caused by polyI: C, indicating that PA potently relieves the symptoms of PBC disease. Meanwhile, PA lowers the indicators of liver damage and liver fibrosis, hinting that PA is efficient in assuaging PBC-induced liver damage and liver fibrosis.

The inflammasome is a protein complex. Induced by diverse endogenous, pathogenic, or environmental red flag, it oligomerizes and activates the caspase-1 cascade to generate IL-1β, as well as IL-18^20^. Inflammasomes have four known structural subsets, including NLR family, NLRP1, NLRP3, NLRC4 and AIM2^21^. NLRP3 is the most investigated inflammasome, containing leucine-rich repeats, NBD, and N-terminal pyridine domain, allowing ASC recruitment to vitalize pro-caspase 1^22^. The complete activation of NLRP3 inflammasome is first launched by diverse PAMPs and DAMPs, resulting in up-regulated IL-1β precursor, IL-18 precursor, as well as inflammasome components, which are then assembled into inflammasome structures and produce pro-inflammatory interleukins^23^^,^ 24. Research has corroborated that NLRP3 can be vitalized by diverse activators, such as oxidative stress, low K ion concentration, bacteria, viruses, lipopolysaccharides, cholesterol crystals, etc.^25^^-^ 29. Injection of polyI: C results in increased MDA content and decreased GSH-px/SOD activity in the liver tissues of mice, then oxidative stress is generated in the body and NLRP3 was activated, posing up-regulated L-1β, IL-18 and caspase-1, as well as the occurrence of inflammation response, thereby causing liver damage and fibrosis and finally the preparation of PBC mouse model.

Studies have illuminated that TNF level, a key cytokine related to liver pathology, is increased in NLRP3-activated mouse liver. NLRP3 inflammasome driven liver injury and fibrosis can be impacted by IL-17 and TNF levels. Among them, the effect of TNF is more pronounced^30^. Inflammasomes are connected to the progress of fibrosis, and the influence of viruses or drugs can activate NLRP3 inflammasomes and subsequently facilitate IL-1β generation. Moreover, IL-1β will contribute to the generation of the primary fibrotic cytokine, namely TGF-β. Increased TGF-β levels can activate epithelial cells and fibroblasts to proliferate and transform into myofibroblasts. This ultimately leads to increased production of collagen, which is a key step in fiber formation. CXC chemokines, another product of IL-1β, is capable of attracting neutrophils to epithelial cells, in which they can not only induce larger damage, but also cause fibrosis^31^. Hence, the pathway leading to the progress of fibrosis and inflammation is concerned with IL-1β. RA curbs NLRP3 formation in PBC mice and inhibits its cascading inflammatory pathway, allaying the degree of liver injury and liver fibrosis in PBC mice.

## Conclusion

PA can moderate PBC-induced liver fibrosis in mice and probably work by muffling the formation of NLRP3.
